# Age-dependent antibody profiles to plasmodium antigens are differentially associated with two artemisinin combination therapy outcomes in high transmission setting

**DOI:** 10.3389/fmed.2022.991807

**Published:** 2022-10-13

**Authors:** Ben Andagalu, Pinyi Lu, Irene Onyango, Elke Bergmann-Leitner, Ruth Wasuna, Geoffrey Odhiambo, Lorna J. Chebon-Bore, Luicer A. Ingasia, Dennis W. Juma, Benjamin Opot, Agnes Cheruiyot, Redemptah Yeda, Charles Okudo, Raphael Okoth, Gladys Chemwor, Joseph Campo, Anders Wallqvist, Hoseah M. Akala, Daniel Ochiel, Bernhards Ogutu, Sidhartha Chaudhury, Edwin Kamau

**Affiliations:** ^1^Department of Emerging and Infectious Diseases (DEID), United States Army Medical Research Directorate-Africa (USAMRD-A), Kenya Medical Research Institute (KEMRI)/Walter Reed Project, Kisumu, Kenya; ^2^Biotechnology High Performance Computing Software Applications Institute, Telemedicine and Advanced Technology Research Center, U.S. Army Medical Research and Development Command, Fort Detrick, MD, United States; ^3^Henry M. Jackson Foundation for the Advancement of Military Medicine Inc., Bethesda, MD, United States; ^4^Biologics Research and Development, Walter Reed Army Institute of Research, Silver Spring, MD, United States; ^5^Antigen Discovery Inc., Irvine, CA, United States; ^6^Kenya Medical Research Institute (KEMRI), Nairobi, Kenya; ^7^Center for Enabling Capabilities, Walter Reed Army Institute of Research, Silver Spring, MD, United States; ^8^U.S. Military HIV Research Program, Walter Reed Army Institute of Research, Silver Spring, MD, United States; ^9^Department of Pathology and Laboratory Medicine, David Geffen School of Medicine, University of California, Los Angeles, Los Angeles, CA, United States

**Keywords:** artemisinin combination therapy, machine learning, modeling, immunoprofiling, malaria, artesunate-mefloquine, artemether-lumefantrine, computational analysis

## Abstract

The impact of pre-existing immunity on the efficacy of artemisinin combination therapy is largely unknown. We performed in-depth profiling of serological responses in a therapeutic efficacy study [comparing artesunate-mefloquine (ASMQ) and artemether-lumefantrine (AL)] using a proteomic microarray. Responses to over 200 *Plasmodium* antigens were significantly associated with ASMQ treatment outcome but not AL. We used machine learning to develop predictive models of treatment outcome based on the immunoprofile data. The models predict treatment outcome for ASMQ with high (72–85%) accuracy, but could not predict treatment outcome for AL. This divergent treatment outcome suggests that humoral immunity may synergize with the longer mefloquine half-life to provide a prophylactic effect at 28–42 days post-treatment, which was further supported by simulated pharmacokinetic profiling. Our computational approach and modeling revealed the synergistic effect of pre-existing immunity in patients with drug combination that has an extended efficacy on providing long term treatment efficacy of ASMQ.

## Introduction

Therapeutic efficacy studies (TESs) are used to monitor efficacy of antimalarial drugs including assessment of clinical and parasitological outcome for artemisinin-based combination therapies (ACTs), the first-line treatment for uncomplicated *Plasmodium falciparum* malaria. TESs conducted at regular intervals in the same location can be used for the detection of the decline of drug efficacy over time. Key indicators monitored during ACTs TESs include proportion of patients who are parasitemic on day 3, and treatment failure by days 28 or 42 (1). Naturally acquired immunity is a key determinant of antimalarial therapeutic response (2), which is highly influenced by transmission intensity (3), and age of the patient (4). Pharmacokinetics (PK) and pharmacodynamics of artemisinin derivatives and partner drugs in ACTs are also important when interpreting TES data.

Artemether-lumefantrine (AL) is the most widely used ACTs in sub-Saharan Africa (sSA), followed by artesunate-amodiaquine (ASAQ) (5). A study that investigated clinical determinants of early parasitological response to ACTs in African patients found that risks of persistent parasitemia on the first and the second day were higher in patients treated with AL compared to those treated with dihydroartemisinin-piperaquine (DP) and ASAQ (6). However, on the third day, the difference was not apparent. Artesunate-mefloquine (ASMQ) has been extensively used in Asia and Latin America but not in sSA because of the availability of other more affordable ACTs (7), concerns for mefloquine resistance seen in Southeast Asia (SEA) (8), and side effects such as excessive vomiting in children (9).

The power of computational tools and mathematical modeling in resolving complex biological questions such as identifying correlates of protection (10–12) or biomarkers of disease (13–16) is now apparent. We have previously used computational integration of immunoprofiling data and modeling to identify immune signature of vaccine adjuvants (10, 17) and vaccine-induced immune correlates of protection (12, 18). In a previous study, we explored the association between the antibody profiles to five Plasmodium antigens and parasite clearance kinetics using part of the TES data presented here (19). Although the scope of this pilot study was limited to five Plasmodium antigens and focused on parasite clearance kinetics in the first 3 days of treatment, it revealed that pre-existing immunity does play a role in treatment outcome thus laying the foundation for the present work. The present, in-depth report on the TES data demonstrates the power of bioinformatics by integrating microarray data and clinical drug and modeling that led to the identification of biomarkers (antigens) associated with a specific treatment outcome within the context of ACTs TES following natural infections.

## Materials and methods

### Study design and participants

This was a randomized, open-label, two-cohort trial, each with two arms conducted in western Kenya, a high transmission, holoendemic region. The study was approved by Institutional Review Boards (IRBs) and Human Subjects Protection Branch, and was registered at clinicaltrials.gov (NCT01976780). Cohort I study was conducted between June 2013 and November 2014, and assessed ASMQ and AL, while cohort II study was conducted between December 2014 and July 2015, and assessed DP and AL. Potential study participants (age 6 months- 65 years) with uncomplicated malaria were identified using malaria rapid diagnostic test (mRDT). Informed consent/assent from the participants, parents or legally authorized representatives was obtained prior to screening procedures. Study details are described in Supplementary material.

### Study procedures

Enrolled participants were randomized for malaria treatments and treated with DP (Duo-cotecxin^®^—Holly Cotec Pharmaceuticals, China), ASMQ or AL (Coartem^®^—Novartis Pharma Ag, Switzerland). Details of drug treatment scheduling, dosing, and administration are provided in Supplementary Method.

During the treatment phase of the studies, blood samples were collected at hours 0, 4, 8, 12, 18, 24, and thereafter, every 6 h until two consecutive negative smears for malaria were obtained. Upon completion of study treatment, participants were followed up weekly from day 7 through day 42.

### Study outcomes

WHO definitions for treatment outcomes in malaria drug efficacy studies were used (1). Parasite clearance rates were calculated using the WWARN, PCE tool located at http://www.wwarn.org/toolkit/data-management/parasite-clearance-estimator. Log transformed parasite density was plotted against time in hours to generate the slope half-life which is defined as the time needed for parasitemia to be reduced by half.

### Laboratory procedures

Malaria microscopy was performed using standardized procedures. *In vitro* drug sensitivity testing was conducted on day 0 (pre-treatment samples) as well as on samples collected from participants who had reappearance of parasites on follow-up visits as previously described (20). Molecular testing was performed as previously described (20).

### Protein microarrays and antibody profiles

A protein microarray containing a total of 1,087 *P falciparum* antigens [3D7 proteome (Antigen Discovery Inc., USA)] was used as previously described (21) to establish the antibody profile of pre-treatment sera for cohort I (ASMQ and AL arms) and perform bioinformatics, data analysis, and modeling. Details on the methodology are provided in Supplementary material.

### Bioinformatics, data analysis, and modeling

Detailed bioinformatics, data analysis, and modeling methods can be found in Supplementary material. Briefly, to identify antibody signal intensities that differed with respect to different treatment outcomes, univariate analysis was conducted for each antibody signal in the immunoprofile. ASMQ and AL study arms were analyzed separately. Within each arm, participants were further classified as treatment success or treatment failure based on non-PCR-corrected Adequate Clinical and Parasitological Response (nPC-ACPR) on day 28 and 42 per World Health Organization (WHO) definition and guidance for the treatment of malaria (1). Each antibody signal was compared between treatment success (nPC-ACPR = 1) and treatment failure (nPC-ACPR = 0). Random forest and logistic regression were applied to build machine learning models using all antibody signals to predict individual participants’ treatment outcome (nPC-ACPR). To evaluate the predictive accuracy of random forest models, cross-validation was utilized, where data samples were subsampled by up-sampling. This aggregation for training and prediction performance was evaluated on data samples that were not used in training. Models’ performance was expressed as both a percentage of correctly predicted outcomes with a Cohen’s kappa value, and as the area under the curve of the receiver operating characteristic (AUCROC). Cohen’s kappa statistic is a measure that can handle imbalanced class problems. A kappa value larger than 0.4 can indicate that the classifier is performing better than a classifier that guesses at random according to the frequency of each class. To assess the statistical significance of the models and check the overfitting that might occur in the machine learning process, AUCROC-based permutation tests were carried out. Relative importance scores of each antibody signal were calculated using random forest models. Principal component analysis (PCA) was applied to all the antibody signals with relative importance scores higher than 50. Antibody signal intensities of participants in the ASMQ arm were plotted using principal component (PC)1 and PC2. We constructed one-compartment PK models for artemether, artesunate, lumefantrine and mefloquine, respectively, using the *linpk* R package. The values of PK parameters, including bioavailable fraction, central clearance, central volume, and first-order absorption rate, were obtained from a previous study (22). All statistical analyses were performed using the R *stats* package and STATA version 13 (StataCorp) while machine learning was carried out using the R *caret* package. The R codes are available in GitHub^1^ and datasets are available on request.

## Results

### Differences in anti-parasitic efficacy of the drug combinations

**FIGURE 1:**
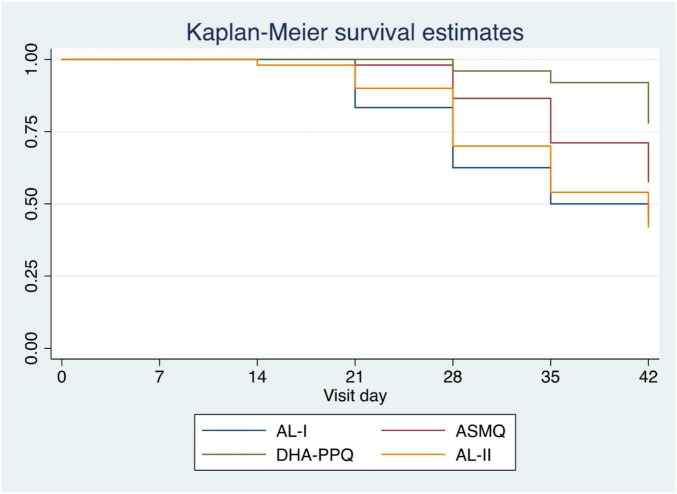
Kaplan-Meier survival estimate for time to malaria infection following ACT treatment. Survival curves are shown for Cohort 1, AL subjects (blue), ASMQ subjects (red) and Cohort II AL subjects (orange), and DHA-PPQ subjects (purple) as a function of days post-treatment.

A total of 236 study participants (*n* = 118 in each cohort) were enrolled of which 200 (84.7%) participants completed 42-day follow-up, 100 from each cohort; 52 in ASMQ arm and 48 in AL arm in cohort I and 50 in each arm in cohort II (Supplementary Figure 1 for study disposition chart). There were no notable differences in the baseline characteristics of the enrolled participants (Supplementary Table 1), and no cases of early treatment failure (Figure 1). All study participants achieved 100% PCR-corrected ACPR (PC-ACPR) rates at day 28 and 42 (Table 1). There were no significant differences in the parasite clearance half-lives between the study arms (Table 2). The maximum parasite clearance slope half-lives observed for both cohorts were 4.2 (AL arm in cohort I) and 4.3 (AL arm in cohort II) hours falling within the WHO recommended cut-off for suspected artemisinin resistance (1). However, PC50 and PC99 data (time for the initial parasite density to fall by 50 or 99%) revealed ASMQ and DP outperformed AL by clearing parasites faster (Table 2). Further, participants who received ASMQ and DP achieved better nPC-ACPR at day 28 and 42 compared to those who received AL (Table 1), with significant difference present in cohort I for ASMQ vs. AL on day 28 (*p* = 0.042) but not on day 42 (*p* = 0.280), and in cohort II, a significant difference was present for DP vs. AL on day 28 (*p* = 0.001) and on day 42 (*p* = 0.008). Of note, none of the study participants developed side effects including those in ASMQ arm.

**TABLE 1:** Rates of adequate clinical and parasitological response (ACPR) with and without PCR corrections.

	ASMQ	AL	Difference% (95% CI)	DP	AL	Difference% (95% CI)
Day 42	***n*** ** = 52**	***n*** ** = 48**		***n*** ** = 50**	***n*** ** = 50**	
PC-ACPR	52 (100%)	48 (100%)		50 (100%)	50 (100%)	
nPC-ACPR	30 (57.7%)	21 (43.8%)	–13.9% (–33.4 to 5.5)	39 (78.0%)	21 (42.0%)	–36% (–53.8 to –18.1)
Day 28	***n*** ** = 53**	***n*** ** = 51**		***n*** ** = 53**	***n*** ** = 51**	
PC-ACPR	53 (100%)	51 (100%)		53 (100%)	51 (100%)	
nPC-ACPR	45 (84.9%)	32 (62.8%)	–22.2% (–38.6 to –5.8)	50 (96.2%)	36 (70.6%)	–25.6% (–39.1 to –12.0)

**TABLE 2:** Parasite clearance rates.

Parameters	ASMQ	AL-I	DP	AL-II

Total analyzed for T_1/2_	58	50	57	53
Slope half-life median (IQR)	2.3 (1.8–2.7)	2.5 (2.2–3.0)	2.2 (1.9–2.5)	2.3 (2.0–2.9)
Slope half-life mean (range)	2.2 (0.98–3.6)	2.6 (1.5–4.2)	2.2 (1.2–3.6)	2.4 (1.4–4.3)
PC50 median (IQR)	4.0 (2.6–6.0)	7.4 (4.8–9.6)	4.1 (3.2–6.0)	6.7 (4.4–8.8)
PC50 mean (range)	4.2 (0.28–11.1)	7.4 (0.5–15.3)	4.6 (0.27–11.3)	6.5 (0.24–11.6)
PC99 median (IQR)	17.0 (13.4–19.2)	21.5 (18.3–24.8)	16.8 (14.6–19.5)	20.1 (17.5–22.1)
PC99 mean (range)	16.6 (6.5–25.6)	21.9 (10.0–33.0)	17.0 (7.3–27.9)	20.0 (9.1–30.5)

Data shows parasite slope half-lives and parasite clearance rates for cohort I (ASMQ and AL-I) and cohort II (DP and AL-II) in hours.

### *In vitro* and molecular analyses

*In vitro* susceptibility testing to AL component drugs (artemether and lumefantrine) was successfully performed in some of the parasite isolates (Supplementary Figure 2). The parasite isolates IC50 values for both drugs remained unchanged, similarly to the published IC50 (17). K13 mutations were present, but none of the K13 mutations identified as markers of artemisinin resistance in SEA. We investigated the polymorphisms in pfcrt (K76) and pfmdr1 (N86, 184F and D1246), and pfmdr1 copy numbers, which are associated with AL selection in sSA parasites (17). The frequencies of these mutations and pfmdr1 copy number variation were similar in both cohorts and to the previously published data (17).

### Wider breadth of humoral immunity confers better non-PCR-corrected adequate clinical and parasitological response outcome in artesunate-mefloquine arm

**FIGURE 2:**
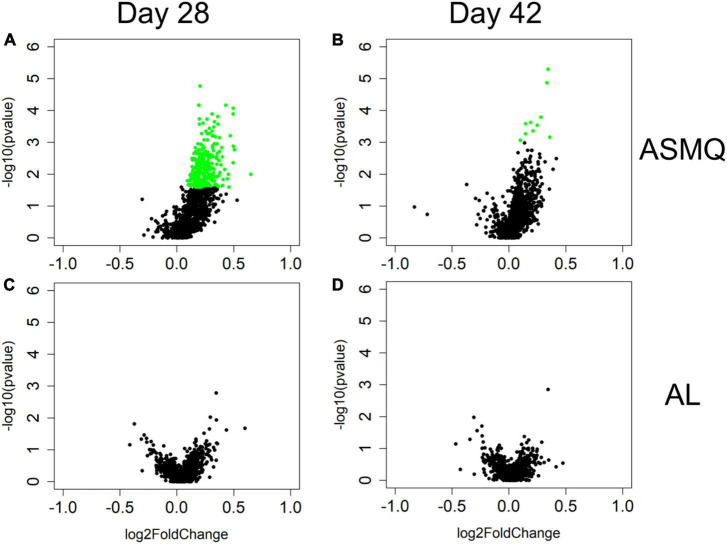
nPC-ACPR-associated differences in subjects’ humoral immunity to malaria in the ASMQ and AL arms. Univariate analyses were applied to identify differences in subjects’ humoral immunity associated with ASMQ outcomes on day 28 **(A)** and day 42 **(B)**, and with AL outcomes on day 28 **(C)** and day 42 **(D)**, respectively. Volcano plots were used to present analysis results. The *x*-axis is log2 ratio of malaria antigen-specific antibody signals of subjects presenting nPC-ACPR to those of subjects not presenting nPC-ACPR. The *y*-axis is *P*-values based on –log10. The green dots represent the antibody responses with *P* < 0.05 and Benjamini-Hochberg adjusted *P* < 0.1.

**FIGURE 3:**
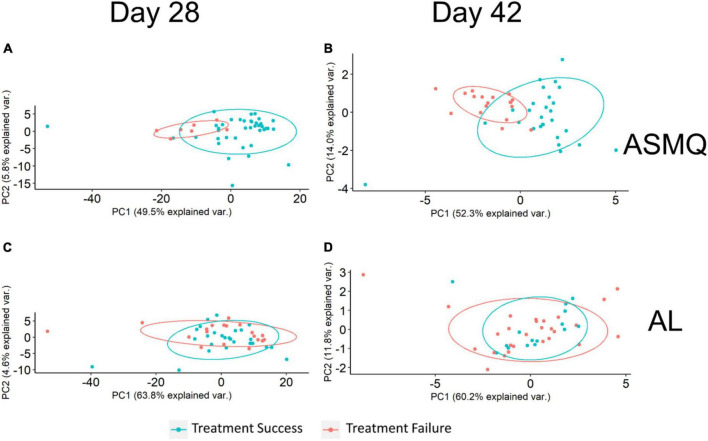
Principal Component Analysis (PCA) plots of treatment outcome-specific differences. **(A,B)**. PCA used antibody signals with adjusted *P* < 0.1 to visualize treatment outcome-specific differences in the ASMQ arm, which were identified by univariate analyses applied to identify differences in subjects’ humoral immunity associated with ASMQ outcomes on day 28 and 42, respectively. **(C,D)**. PCA used the same antibody signals as **(A,B)** to visualize treatment outcome-specific differences in the AL arm.

Since the variances in the response to drug treatment were not due to differences in parasite genetic diversity profiles, we sought to determine whether distinct immunoprofiles in participants treated with the different drug combinations impacted the clinical and parasitological outcome in cohort I. Although there were no significant differences in the parasite clearance half-lives between the study arms regardless of the treatment used, the parasite clearance kinetics based on PC50/PC99 clearly demonstrated there was a lag phase in AL treatment compared to ASMQ and DP (Table 2). We used nPC-ACPR as endpoint data to dichotomize study participants’ response to treatment regardless of whether it was reinfection or recrudescence because it indicated differences in the ability of the study participants to control parasites likely due to existing anti-Plasmodial antibodies. Microarrays analyses to establish the Plasmodium-specific antibody profiles were successfully performed for 91 (46 in ASMQ and 45 in AL) of the 104 participants who completed day 28 follow-up, and 87 (45 in ASMQ and 42 in AL) of 100 participants who completed day 42. We carried out univariate analyses to compare the antibody profiles established by the microarrays between participants in ASMQ and AL arms who achieved nPC-ACPR vs. those who did not, on day 28 and 42. Significant nPC-ACPR associated differences were present in the ASMQ arm (Figures 2A,B), but not in the AL arm (Figures 2C,D). In the ASMQ arm, antibody responses to 277 antigens with *P* < 0.05 and adjusted *P* < 0.1 (Figure 2A) were significantly different between participants that showed nPC-ACPR on day 28 compared to those who did not. On day 42, significant differences were observed for antibody responses to 10 antigens with *P* < 0.05 and adjusted *P* < 0.1 (Figure 2B). The right-skewed pattern in the volcano plots (Figures 2A,B) indicates that participants maintaining nPC-ACPR in the ASMQ arm had higher humoral immunity to *P falciparum* antigens compared those who did not. The specific antigens that correspond to these antibody responses are listed in Supplementary Table 2, ranked by corresponding Benjamini-Hochberg adjusted *P*-values. To determine whether distinct immunoprofiles are associated with parasitological outcome, microarray and treatment outcome data were integrated and a PCA was performed. In the ASMQ arm, participants clustered separately based on their treatment outcome, showing clear systematic differences in immune responses (Figures 3A,B). However, there was no separation in the AL arm (Figures 3C,D).

### Antibody responses associated with non-PCR-corrected adequate clinical and parasitological response are age dependent

**FIGURE 4:**
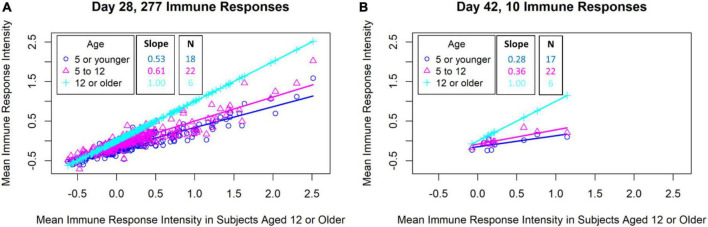
Comparison of malaria antigen-specific antibody responses of subjects in different age groups. The mean antigen-specific antibody signal intensities to *P falciparum* proteins (identified through univariate analyses) from subjects in varying ages (*y*-axis) were plotted against the mean intensities for the oldest subject group (*x*-axis). **(A)** Scatterplot of mean antibody signal intensities of 277 *P falciparum* proteins associated with ASMQ outcomes on day 28. **(B)** Scatterplot of mean antibody signal intensities of 10 *P falciparum* proteins associated with ASMQ outcomes on day 42.

To investigate the magnitude of immune responses with age, we compared antibody signal intensities of the study participants in different age groups (5 or younger, 5–12, and 12 or older) in the ASMQ arm. Normalizing the mean signal intensities to *P falciparum* proteins from participants at varying ages against the mean intensities for the oldest study participant group revealed that the magnitude of antibody responses to these antigens increased with age (Figure 4). Among antibody responses associated with nPC-ACPR on day 28, children (participants < 12 years) showed approximately half the magnitude of antibody responses as older participants (≥12 years), as indicated by the slope. This effect was more pronounced in antibody responses associated with nPC-ACPR on day 42, where children showed approximately a third of the magnitude of responses as older participants.

### Machine learning can predict treatment outcome for artesunate-mefloquine using humoral immunity data

**FIGURE 5:**
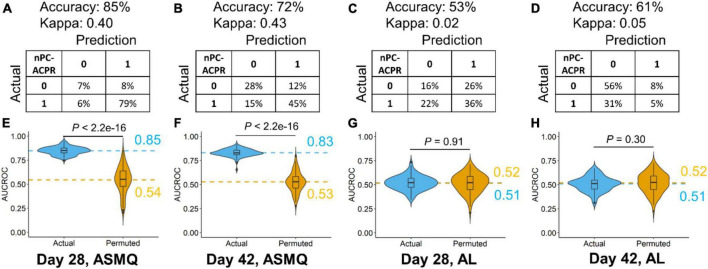
Performance evaluation of random forest models predicting ASMQ outcomes on day 28 and 42 **(A–D)**. Prediction accuracy, kappa, and confusion matrices. The rows of confusion matrices represent the actual treatment outcomes, whereas the columns indicate the predicted treatment outcomes **(E–H)**. Comparison of AUCROC values from 100 repetitions of 100 times repeated fivefold cross-validation using actual (blue) vs. permutated (yellow) nPC-ACPR labels. Dashed line represented the mean AUCROC values. Significance is determined using Mann-Whitney-Wilcoxon test.

Machine learning methods (random forest models confirmed by logistic regression models) were used to assess the degree to which humoral immunity to malaria could predict treatment outcome. For the ASMQ arm, 100 random forest models were built for predicting treatment outcome using antibody signals that were significantly different between participants that showed nPC-ACPR compared to those who did not. Models built for predicting treatment outcomes on day 28 achieved 85% accuracy (Kappa: 0.40) with an average AUCROC of 0.85 (Figures 5A,E), and on day 42, the accuracy was 72% (Kappa: 0.43) with an average AUCROC of 0.83 (Figures 5B,F). Randomly shuffled nPC-ACPR outcomes across participants to remove any possible link between humoral immunity and outcome were used as negative control for the machine learning analysis to test for overfitting. The average AUCROC for using the randomly shuffled data for day 28 and 42 in ASMQ arm were 0.54 and 0.53, which were significantly lower than the average AUCROC of actual models (Figures 5A,B). In the AL arm, machine learning models could not predict nPC-ACPR outcome on day 28 or 42, achieving accuracies of 53% (Kappa: 0.02) and 61% (Kappa: 0.05), respectively, with an average AUCROC of 0.51, indicating an accuracy no better than random chance (Figures 5C,D,G,H). Relative importance scores of each antibody signal were calculated only for the ASMQ arm using random forest models, which could not only help get a better understanding of the model’s logic, but also help identify antibody signals more related to the therapeutic efficacy of ASMQ (Supplementary Table 3).

### Simulation of artesunate-mefloquine and artemether-lumefantrine pharmacokinetics profiles

**FIGURE 6:**
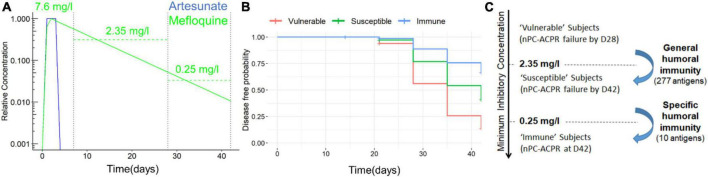
PK profile of ASMQ and estimation of MIC of mefloquine. **(A)** Simulated PK profile of ASMQ for artesunate (blue) and mefloquine (green). Estimated peak concentration, and average concentration between day 7 and 28, and day 28 and 42 are shown for mefloquine are labeled. **(B)** Estimated disease-free probability for all ASMQ subjects classified as “susceptible,” “intermediate,” and “immune” based on humoral immunity using the day 28 and 42 predictive models. **(C)** Relationship between humoral immunity and MIC of mefloquine based on ACPR outcome, univariate analysis, and simulated PK profiles.

To explore how humoral immunity and treatment interact, we simulated PK profile of ASMQ and AL out to day 28 and 42. The PK profiles of both regimens show that the concentration of artemisinin derivative drugs clears within 6 days post-treatment. In the AL regimen, lumefantrine is cleared by day 11 post-treatment, therefore is unlikely to have a major impact on day 28 and 42 outcomes (Figure 6A and Supplementary Figure 3). By comparison, in the ASMQ regimen, mefloquine has a much longer apparent half-life, with concentrations relative to peak concentration of 30.9% at day 28, and 3.3% at day 42 (Figure 6A), representing 3- and 30-fold reductions from the peak concentrations.

Based on this data we formulated the hypothesis that humoral immunity augments the efficacy of mefloquine when it is at sub-therapeutic concentrations. The minimum inhibitory concentration (MIC) of the drug required to be effective is likely to vary between individuals. Given that all participants showed nPC-ACPR at day 7, for participants that showed nPC-ACPR failure by day 28, it is plausible the drug concentration fell below individual’s MIC between day 7 and 28. Based on our simulated PK profiles, the average mefloquine concentration during that time span was 2.35 mg/l. Likewise, for participants that showed nPC-ACPR at day 28, but nPC-ACPR failure by day 42, the mefloquine concentration fell below their MIC within the time span of day 28 and 42, during which an average concentration was estimated to be 0.25 mg/l. Therefore, based on our estimates, “vulnerable” participants who had nPC-ACPR failure by day 28 have an average MIC > 2.35 mg/l, while immune participants who show nPC-ACPR even out to day 42 have an average MIC of < 0.25 mg/l. In addition, Cox regression analysis showed a strong association (*P* < 0.01) between the time to malaria re-infection and classes of participants identified by machine learning models (Figures 6B,C).

## Discussion

Our study provides crucial findings on the efficacy of ACTs in treatment of uncomplicated *P falciparum* in high transmission settings. To the best of our knowledge, this is the first study to demonstrate divergent impact of serological immune profiles on treatment outcomes based on ACT treatment using a computational approach. Machine learning approaches identified *P falciparum* antigens that are highly predictive of successful ASMQ treatment outcome. Our modeling data suggest that at sub-therapeutic concentrations, mefloquine acts synergistically with Plasmodium-specific antibody responses to provide extended protection against clinical and parasitological failure. In sSA, there is a need to deploy additional ACTs or new class of antimalarial drugs to avoid development of resistance to the current first-line treatments. By identifying specific antigens associated with, and predictive of treatment outcome for specific antimalarial drugs, our data support the notion of smart deployment of new ACTs and other antimalarial drugs, where decisions are informed by individual and population immune profiles, and therefore strategically prioritized for each region or a country.

The high efficacy of all the ACTs tested in this study can be attributed to high transmission rates and prevalent immunity. We showed ASMQ and DP outperformed AL on day 28, as well as day 42, corroborating other studies (6, 23). This has previously been attributed to the long half-lives of mefloquine and piperaquine, which are thought to provide 4–6 weeks prophylaxis after treatment compared to 3–4 days for lumefantrine (24). Using PK modeling, we estimated mefloquine concentrations to be at 30% of peak concentration on day 28, while lumefantrine was completely cleared by 14 days, confirming the persistence of mefloquine and its role in the apparent post-treatment prophylaxis observed in the ASMQ cohort.

Host immunity is an important determinant of treatment outcome in *P falciparum* malaria infections (2), with the magnitude of immunological response increasing with age (4). In this study, we show that the interaction between humoral immunity and residual mefloquine concentration is important in providing protection and predicting treatment outcome. This is supported by the following observations: First, if humoral immunity alone was sufficient for nPC-ACPR out to day 28 and 42, then immunity would have predicted protection in AL arm as well; second, if residual mefloquine concentration alone was sufficient to determine nPC-ACPR outcome, then humoral immunity wouldn’t have predicted outcome in ASMQ; and third, immunity is likely the only explanation for differences in nPC-ACPR based on age, as age-specific differences in PK profiles of AL and ASMQ have not been reported. Machine learning identified three classes of patients (vulnerable, susceptible, and immune) in the ASMQ arm based on immune data (Figure 6B). Our findings suggest that general humoral immunity to a wide range of Plasmodium antigens is sufficient to provide protection in the presence of residual mefloquine concentrations out to day 28, while specific immunity to a handful of select antigens is necessary to provide protection in very low residual mefloquine concentrations out to day 42.

Reports of mefloquine side-effects including early vomiting, mental and neurological concerns might be contributing to the poor scale-up of ASMQ in Africa (9, 23, 25). Dosing and timing of when mefloquine is administered as a combination therapy is important, impacts drug efficacy, and the side effects experienced by the patients. Ter Kuile et al., showed that in children ≤ 2 years, vomiting was reduced by 40% when mefloquine dose of 25 mg/kg was split over 2 days, and by 50% when given on the second day (9). By administering artesunate first, and then mefloquine 24 h later, this reduced vomiting because the patients had recovered clinically and were more likely to tolerate mefloquine. Further, delaying the dose of mefloquine for 24 h after artesunate administration increases mefloquine oral bioavailability substantially probably due to rapid clinical improvement (26). In our study, administration of three doses of artesunate in the first 48 h, and then mefloquine at 72 and 96 h eliminated vomiting and dramatically reduced side effects. As a fixed-dose or non-fixed-dose combination therapy, ASMQ is given over a 3-day period once or twice daily (25, 27–29). Since fixed-dose medication improved compliance, we propose creation of a fixed-dose ASMQ combination that delivers mefloquine after the first 24 or 48 h to allow ample time for clinical recovery of the patient.

This study has limitations: (1) relatively small sample size; our univariate analysis statistical test accounts for sample size and utilizes a multiple test correction to account for the large number of parameters being measured. The immune correlates of outcome were identified in ASMQ cohorts, but not in AL cohorts. This would not have been possible in an under-powered study. (2) The small number of correlates identified in the ASMQ Day 42 cohort could be the result of the waning impact of immunity over time, post-treatment, as the mefloquine concentration decreases. In the future, it will be important to repeat these analyses using other ACTs especially DP due to its high efficacy and the long prophylactic life-span of piperaquine.

## Conclusion

In conclusion, we have demonstrated that data integration, machine learning, and modeling provide a comprehensive approach capturing the underlying complexity of malaria control in sSA. Further, we have shown ASMQ is a highly effective drug, making it an appropriate choice of possible first-line treatment in western Kenya, a region which account for most malaria transmission in the country.

## Data availability statement

The original contributions presented in this study are included in the article/Supplementary material, further inquiries can be directed to the corresponding author.

## Ethics statement

The studies involving human participants were reviewed and approved by the Kenya Medical Research Institute Scientific and Ethical Review Unit (KEMRI-SERU)—KEMRI SSC numbers 2518 and 2722, as well as the Walter Reed Army Institute of Research Institutional Review Board (WRAIR IRB)—WRAIR numbers 1935 and 1935A. The patients/participants provided their written informed consent to participate in this study.

## Author contributions

BA: study principal investigator (PI), funding, data analysis, manuscript draft writing, and reviewing. PL: bioinformatics analysis and draft review. IO: study oversite and recruitment of study participants. EB-L: data analysis, draft writing, and reviewing. RW: study pharmacists. GO, LC-B, LI, DJ, BenO, AC, RY, CO, RO, and GC: laboratory analysis of specimens. JC: immunological data analysis, funding, and manuscript review. AW: bioinformatics analysis and team lead. HA: specimen and personnel management, data analysis, and draft review. DO: data analysis and manuscript review. BerO: consultation and manuscript review. SC: bioinformatics analysis, draft writing, and reviewing. EK: study and team management, concept refinement, draft writing, and reviewing. All authors contributed to the article and approved the submitted version.

## Footnotes

1
https://github.com/BHSAI/immstat2

## Author disclaimer

Material has been reviewed and approved for public release with unlimited distribution by the Walter Reed Army Institute of Research and The Henry M. Jackson Foundation for Advancement of Military Medicine, Inc. (HJF). This manuscript has been approved for public release with unlimited distribution. The investigators have adhered to the policies for protection of human subjects as prescribed in AR 70–25. EB-L, SC, and EK were government employees, and this work was prepared as part of their official duties. Title 17 U.S.C. §105 provides that copyright protection under this title is not available for any work of the U.S. Government. The opinions or assertions contained herein are the private views of the author, and are not to be construed as official, or as reflecting true views of the Department of the Army, the U.S. Department of Defense, or HJF.
