# Supplementary material for: Age-dependent antibody profiles to plasmodium antigens are differentially associated with two artemisinin combination therapy outcomes in high transmission setting
Source: Front Med (Lausanne). 2022 Oct 13;9:991807. doi: 10.3389/fmed.2022.991807 (PMC9606348; doi:10.3389/fmed.2022.991807)
Supplement: Supplementary file 1 [file Data_Sheet_1.pdf]

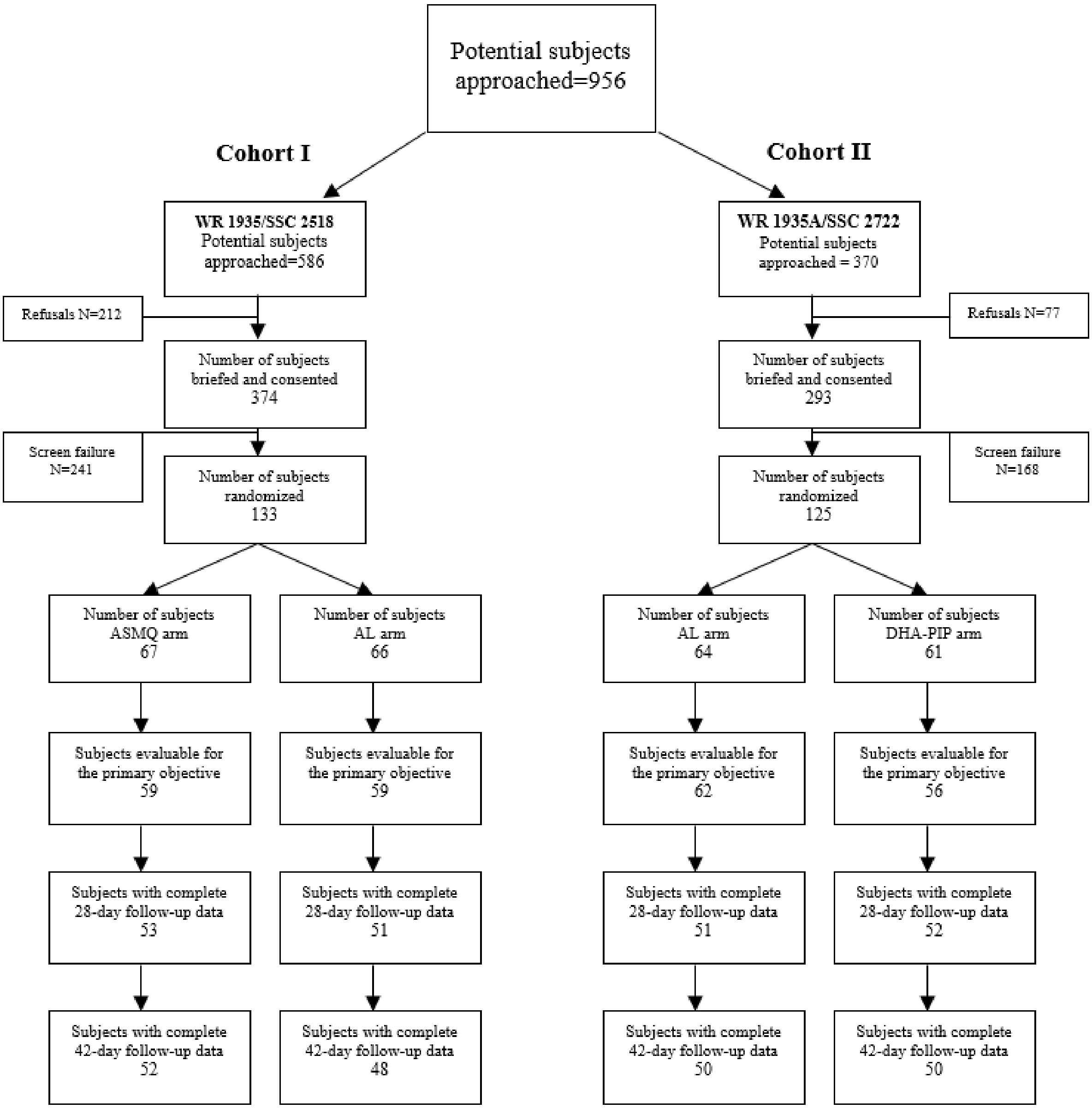

Supplementary Figure 1. Consort Figure.

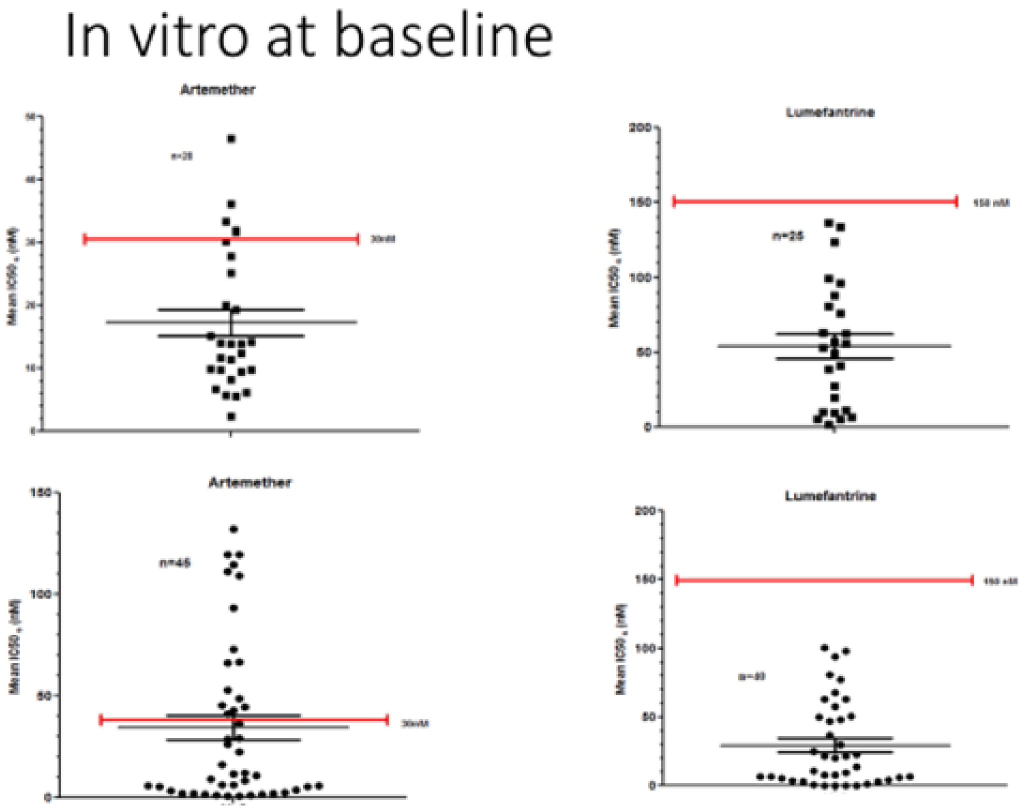

Supplementary Figure 2: In vitro susceptibilities of four malaria drugs against field isolates collected during the efficacy studies. The 50% inhibition concentration in nanomolar are plotted against the study. The red line represents the literature resistance thresholds for drugs. The top row is AL data from cohort I and bottom row is data from cohort II.

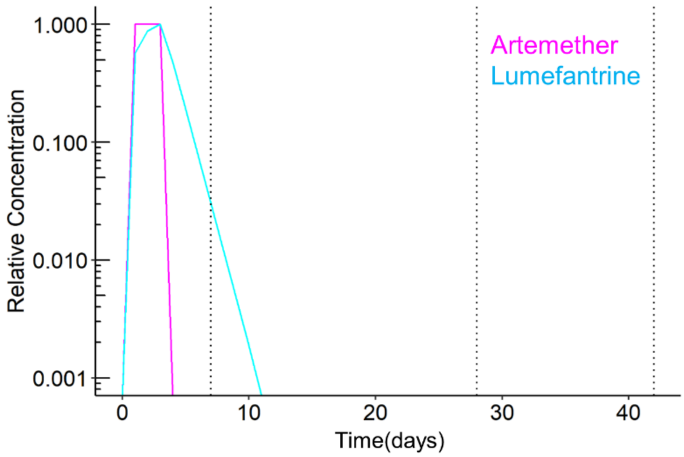

Supplementary Figure 3: Simulated PK profile of AL for artemether (magenta) and lumefantrine (cyan).

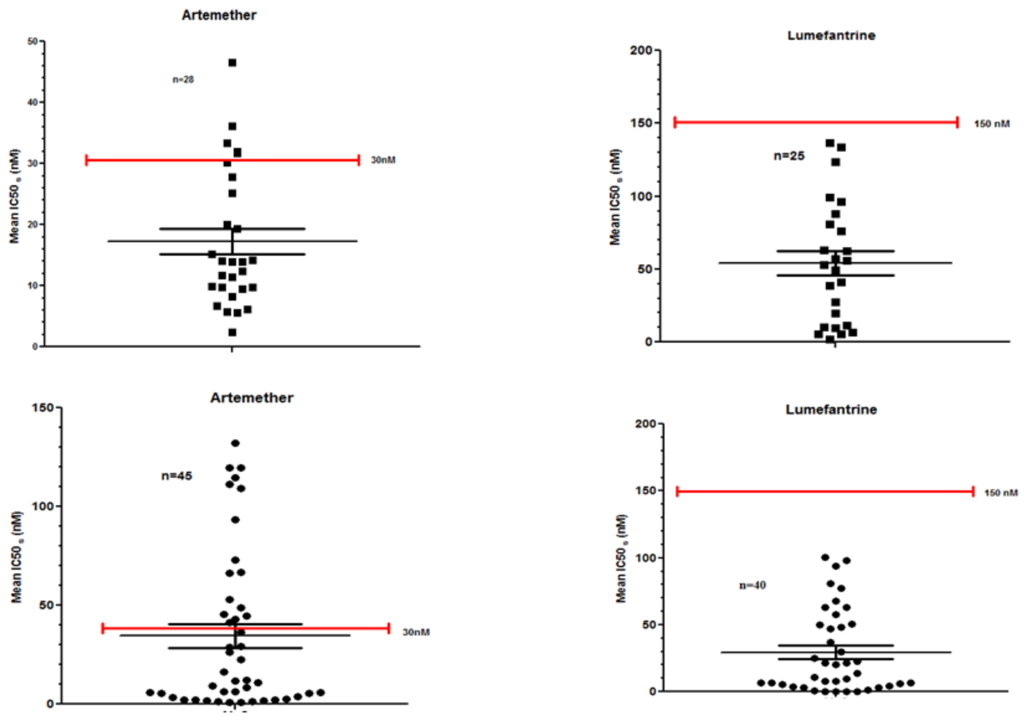

Supplementary Figure 4. In vitro susceptibilities of four malaria drugs against field isolates collected during in vivo I or II efficacy studies. The 50 percent inhibition concentration in nanomolar are plotted against the study. The red line represents the literature resistance thresholds for drugs. The top row is AL data from cohort I and bottom row is data from cohort II**.**

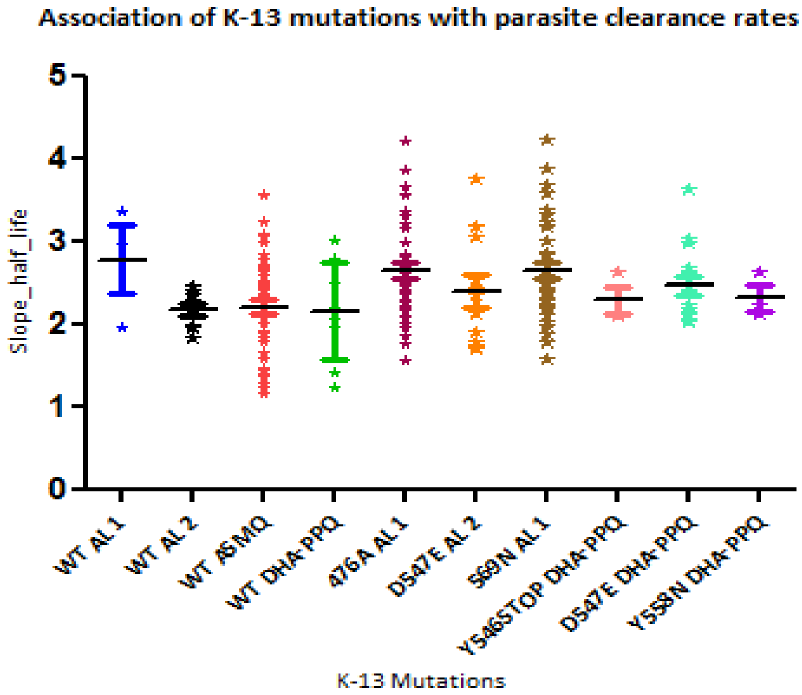

Supplementary Figure 5. Data showing slope half-life correlation with K13 mutations.

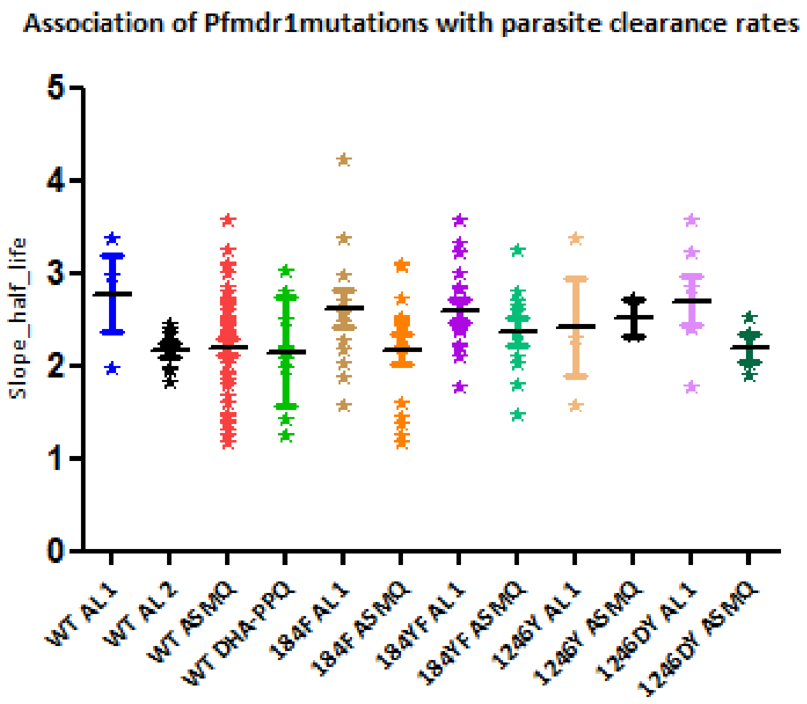

Supplementary Figure 6. Data showing slope half-life correlation with pfcrt and pfmdr1 mutations.
