# Supplementary material for: Age-dependent antibody profiles to plasmodium antigens are differentially associated with two artemisinin combination therapy outcomes in high transmission setting
Source: Front Med (Lausanne). 2022 Oct 13;9:991807. doi: 10.3389/fmed.2022.991807 (PMC9606348; doi:10.3389/fmed.2022.991807)
Supplement: Supplementary file 2 [file Table_1.pdf]

Supplementary Table 1: Baseline characteristics of cohort I (ASMQ and AL-I) and cohort II (DP and AL-II) study participants

	AL - I N=59	ASMQ N=59	AL - II N=62	DP N=56	Overall N=236
Age – mean years (SD)	6.7 (7.1)	8.9 (10.1)	7.0(3.8)	7.3 (3.6)	7.5 (6.7)
Age – median years (range)	5 (1 – 50)	6 (1 – 51)	6 (1 – 22)	7 (2 – 19)	6 (1 – 51)
Sex – female N(%)	34 (57.6%)	35 (59.3%)	31 (50%)	27 (48.2%)	127 (53.8%)
Mean weight kg (SD)	24.0 (15.7)	25.0 (15.1)	22.2 (10.0)	22.1 (8.7)	23.3 (12.7)
Weight range	10.6 – 90.4	11.0 – 76.6	11.0 – 69.4	11.4 – 64.2	10.6 – 90.4
Temperature – mean ^{0}C (range)	38.0 (36.0 – 40.1)	37.5 (36.1 – 40.0)	37.7 (36.0 – 40.1)	37.5 (35.9 – 39.6)	37.7 (35.9 – 40.1)
Mean Hb g/dl (range)	10.6 (7.0 – 15.1)	10.8 (7.2 – 15.8)	11.0 (6.8 – 15.3)	11.0 (6.8 – 15.3)	10.9 (6.6 – 15.8)
Asexual Parasite density – geometric mean per µL (95% CI)	38759 (26194 – 57351)	32789 (21907 – 49078)	36928 (25438 – 53607)	38652 (24505 – 60964)	36709 (30080 – 44799)
Gametocyte carriage N(%)	51(86.4%)	52 (88.1%)	46 (74.2%)	49 (87.5%)	198 (83.9%)
