# Supplementary material for: Age-dependent antibody profiles to plasmodium antigens are differentially associated with two artemisinin combination therapy outcomes in high transmission setting
Source: Front Med (Lausanne). 2022 Oct 13;9:991807. doi: 10.3389/fmed.2022.991807 (PMC9606348; doi:10.3389/fmed.2022.991807)
Supplement: Supplementary file 5 [file Table_4.pdf]

Supplementary Table 4: Adverse events.

	ASMQ	AL	DP	AL
**Number of patients**	**n = 59**	**n = 59**	**n = 62**	**n = 56**
**Anemia**	15(25.4%)	9(15.3%)	7(11.3%)	10(17.9%)
**Diarrhea**	0	0	1(1.6%)	0
**Abdominal pain**	1(1.7%)	0	1(1.6%)	0
**Gastroenteritis**	1(1.7%)	0	2(3.2%)	0
**Tinea capitis**	5(8.5%)	3(5.1%)	4(6.5%)	4(7.1%)
**Respiratory tract infection**	3(5.1%)	15(25.4%)	12(19.4%)	9(16.1%)
**Rash**	2(3.4%)	1(1.7%)	0	3(5.4%)
**Dizziness**	1(1.7%)	0	0	0
**Fatigue**	0	1(1.7%)	0	0
**Headache**	1(1.7%)	1(1.7%)	5(8.1%)	1(1.8%)
**Fever**	0	0	1(1.6%)	0
**Nausea**	1(1.7%)	0	0	0

Some of the adverse events that occurred in study participants. None of the study participant vomited, all adverse events were mild, at low frequency and resolve spontaneously.
