# Supplementary material for: Age-dependent antibody profiles to plasmodium antigens are differentially associated with two artemisinin combination therapy outcomes in high transmission setting
Source: Front Med (Lausanne). 2022 Oct 13;9:991807. doi: 10.3389/fmed.2022.991807 (PMC9606348; doi:10.3389/fmed.2022.991807)
Supplement: Supplementary file 6 [file Table_5.pdf]

Supplementary Table 5: Percentage of *P falciparum* protein features with mass spectrometry evidence for expression during each of the various parasite lifecycle stages.

Parasite Stage	Percent of *P falciparum* proteins targeted by 277 selected Ab immune responses, Day 28 (p-value)	Percent of *P falciparum* proteins targeted by 10 selected Ab immune responses, Day 42 (p-value)	Percent of 1,087 *P falciparum* proteins on microarrays
Liver	14.9 (0.13)	10.0 (0.63)	12.8
Sporozoites	43.5 (0.03)	30.0 (0.42)	38.6
Merozoites	26.1 (0.41)	30.0 (0.53)	26.8
Trophozoites	31.2 (0.48)	60.0 (0.05)	30.8
Gametocytes	37.7 (0.16)	30.0 (0.51)	35.1
